# Characterizing recurrent infections after one-stage revision for periprosthetic joint infection of the knee: a systematic review of the literature

**DOI:** 10.1007/s00590-023-03480-7

**Published:** 2023-03-03

**Authors:** Francesco Bosco, Giorgio Cacciola, Fortunato Giustra, Salvatore Risitano, Marcello Capella, Daniele Vezza, Luca Barberis, Pietro Cavaliere, Alessandro Massè, Luigi Sabatini

**Affiliations:** 1 Centro Traumatologico Ortopedico (CTO), Department of Orthopaedic Surgery, University of Turin, Via Gianfranco Zuretti, 29, 10126 Turin, Italy; 2 Istituto Ortopedico del Mezzogiorno d’Italia “Franco Scalabrino”, 98100 Messina, Via Consolare Pompea Italy; 3 Department of Orthopaedics and Traumatology, Ospedale San Giovanni Bosco - ASL Città di Torino, Piazza del Donatore di Sangue, 3, 10154 Turin, Italy

**Keywords:** One-stage, Total knee replacement, Total knee arthroplasty, TKA, TKR, Periprosthetic joint infection, PJI

## Abstract

**Background:**

Periprosthetic joint infection (PJI) of the knee represents a severe complication after 1.5% to 2% of primary total knee replacement. Although two-stage revision was considered the gold-standard treatment for PJI of the knee, in the last decades, more studies reported the outcomes of one-stage revisions. This systematic review aims to assess reinfection rate, infection-free survival after reoperation for recurrent infection, and the microorganisms involved in both primary and recurrent infection.

**Material and methods:**

A systematic review of all studies reporting the outcome of one-stage revision for PJI of the knee up to September 2022, according to PRISMA criteria and AMSTAR2 guidelines, was performed. Patient demographics, clinical, surgical, and postoperative data were recorded. PROSPERO ID: CRD42022362767.

**Results:**

Eighteen studies with a total of 881 one-stage revisions for PJI of the knee were analyzed. A reinfection rate of 12.2% after an average follow-up of 57.6 months was reported. The most frequent causative microorganism were gram-positive bacteria (71.1%), gram-negative bacteria (7.1%), and polymicrobial infections (8%). The average postoperative knee society score was 81.5, and the average postoperative knee function score was 74.2. The infection-free survival after treatment for recurrent infection was 92.1%. The causative microorganisms at reinfections differed significantly from the primary infection (gram-positive 44.4%, gram-negative 11.1%).

**Conclusion:**

Patients who underwent a one-stage revision for PJI of the knee showed a reinfection rate lower or comparable to other surgical treatments as two-stage or DAIR (debridement, antibiotics, and implant retention). Reoperation for reinfection demonstrates a lower success compared to one-stage revision. Moreover, microbiology differs between primary infection and recurrent infection.

**Level of evidence** Level IV.

## Introduction

Knee periprosthetic joint infection (PJI) is a severe complication that occurs in 1.5–2% of cases after primary total knee replacement (TKR) [1–5]. Although thorough diagnosis remains the cornerstone of PJI management, surgical planning and accurate adherence to treatment principles are essential [6]. Different surgical strategies may be adopted, such as debridement, antibiotics, implant retention (DAIR), and replacement arthroplasty in one or two stages. Furthermore, salvage options for patients with multiple treatment failures, including knee arthrodesis or above-knee amputation (AKA), should be considered [7, 8].

In recent decades, two-stage revision, based on all prosthetic implants’ removal and articular spacer implantation, has been considered the gold-standard treatment for knee PJI [9]. This procedure allows both local antibiotics administration in a concentration above the minimum effective concentration for approximately two weeks and adequate soft tissue tension during the interval period. First introduced in the early 1980s by John Insall, it should provide a higher eradication rate than other surgical solutions [10–12]. However, two-stage revision is associated with longer hospitalization, higher costs, and poor quality of life during the interval period [13]. Furthermore, there is no high-level evidence demonstrating that this technique has a higher success rate than the one-stage procedure [14].

One-stage revision has become increasingly popular recently and should be considered a viable option for chronic PJI treatment in a selected patient group [9]. This surgical procedure presents potential advantages such as reduced morbidity and mortality, hospital length and related costs, and improved quality of life while providing an optimal infection eradication rate [15]. Identifying a known sensitivities microorganism is mandatory; however, conditions like culture-negative PJI or the presence of systemic sepsis signs discourage one-stage revision indication [15, 16].

This systematic review aims to analyze the reinfection rate, survivorship after reoperation for recurrent infection, and the microorganisms involved in both index and recurrent infection in patients undergoing one-stage revision for knee PJI to assist orthopedics in daily practice PJI management.

## Materials and methods

### Research question

A systematic review of the current literature was performed according to the Preferred Reporting Items for Systematic Reviews and Meta-Analyses (PRISMA) and a Measurement Tool to Assess Systematic Reviews (AMSTAR2) guidelines [17]. Medline, Cochrane Library, EMBASE, Scopus, and Web of Science databases were systematically reviewed until September 1, 2022. The following keywords were used in association with the Boolean AND/OR operator to identify relevant studies on one-stage revision TKA: “one-stage”; “single-stage”; “periprosthetic joint infection”; “PJI”; “total knee arthroplasty”; “TKA”; “total knee replacement”; “TKR.”

### Inclusion and exclusion criteria

The inclusion criteria of the studies analyzed were “articles with patients treated with one-stage revision for treatment of knee PJI; with at least ten patients, a minimum follow-up of one year, reporting infection-free survival for one-stage revision; and studies written in English.” “Case reports, biochemical and in vitro studies, reviews, editorials, book chapters, or instructional courses were excluded from the systematic review.”

### Methodological quality assessment

Each article’s levels of evidence (LoE) was recorded using the Oxford Centre for Evidence-Based Medicine criteria [18]. The methodological quality of the studies included was evaluated through the methodological index for non-randomized studies (MINORS) criteria [19–21]. The MINORS score ranges from 0 to 18 for non-comparative studies and 0 to 24 for comparative studies, with a higher score reflecting higher quality. The present systematic review was registered in PROSPERO, ID: CRD42022362767.

### Search strategy and study screening

**Fig. 1:**
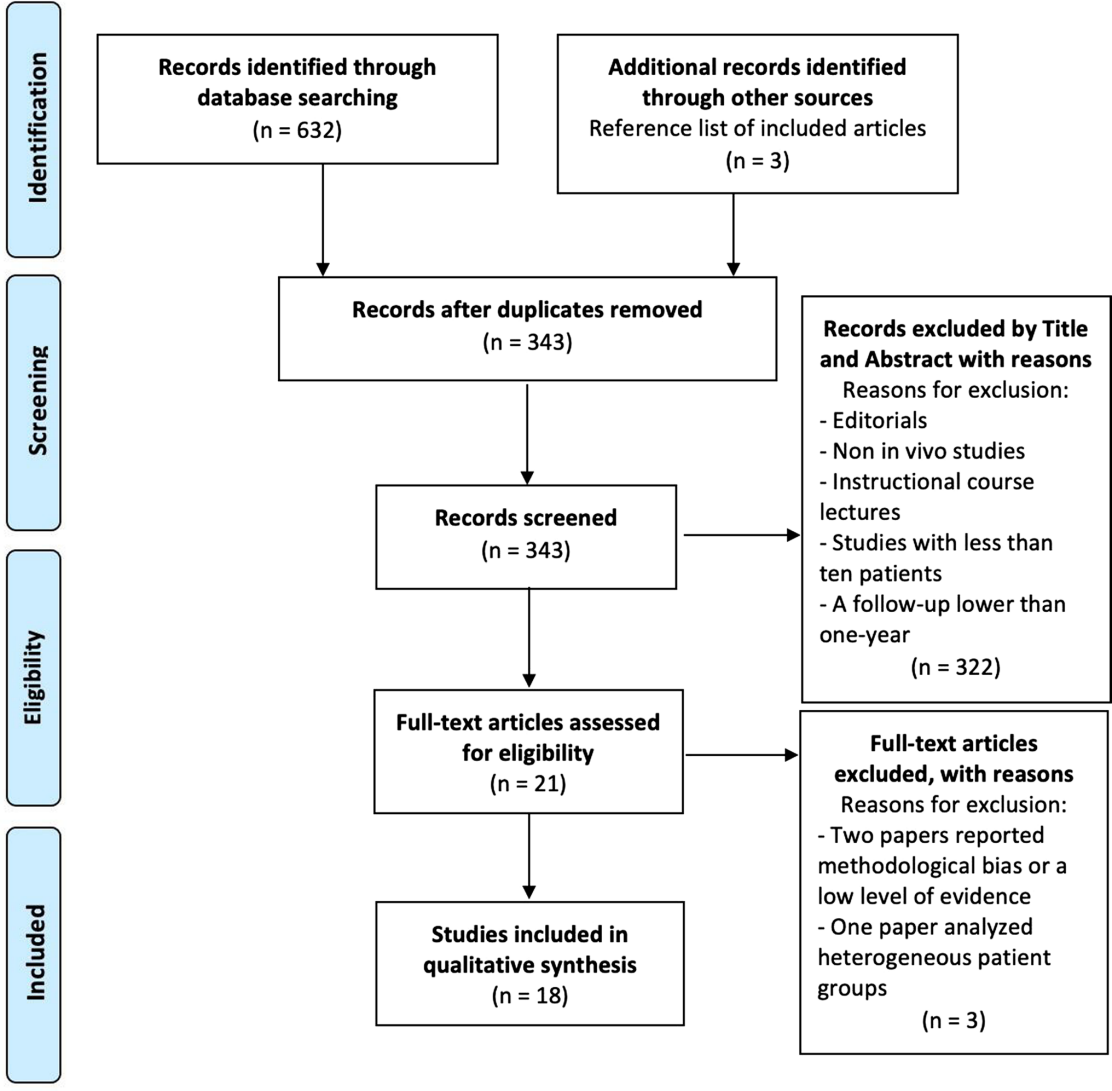
Preferred Reporting Items for Systematic review and Meta-Analysis (PRISMA) flow diagram of studies included in the systematic review

A total of 632 studies were identified through the databases used. After excluding duplicates, 343 studies were included, and after evaluation of the title and abstract, 21 studies were analyzed. After assessing the eligibility of the full-text articles according to the inclusion and exclusion criteria and screening the bibliography of each article to find additional relevant publications, 18 clinical studies were selected and included in this systematic review [22–39]. The PRISMA flowchart with the research strategy is shown in Fig. 1 [17].

### Data extraction

Two reviewers (GC and LB) collected data from the selected studies and inserted them into a standard template. The following study characteristics were extracted: author and year of publication, study characteristics and patient demographics, a summary of reinfection rate after one-stage revision, causative microorganism at the one-stage revision, causative microorganism at reinfection, differences in microbiology between one-stage revision and reinfection. When a study included total hip and knee results of one-stage revision, only data about TKA were collected. When a study included data on different surgical procedures such as DAIR or two-stage revision, only results about one-stage revision were collected.

### Statistical analysis

Statistical analysis was performed with R software, version 4.0.5 (2020; R Core Team, Vienna, Austria). Descriptive statistical analysis was performed for all data extracted by the included studies. Mean values with a measure of variability as standard deviation (SD) or range (minimum–maximum) were calculated for continuous variables. The absolute number, frequency distribution, and Chi-square test were used to analyze categorical variables. A *p* value < 0.05 was considered statistically significant.

## Results

### Study characteristics

Eighteen clinical studies [22–39] were included in the final analysis (Fig. 1) [17]. The LoE and MINORS values for each study are listed in Table 1. Initially, 991 one-stage revisions for knee PJI were included in the analysis. After excluding 104 (10.5%) one-stage revisions due to missing data and patients lost to follow-up, 887 one-stage revisions with a mean age of 67.9 ± 2.9 years were included in the final analysis. The mean follow-up was 57.6 ± 31.8 months. Seventeen studies [17–32, 34–39] reported sex data, and 43.3% were men, and 56.7% were women. The demographic data of the included studies are listed in Table 1.

**Table 1:** Study characteristics and patient demographics

Author and publication year	Study quality		N° of knees, initial cohort/final cohort	N° of knee lost to follow-up and/or died	Age	Male	Female	Follow-up
	LoE	MINOR score	N° / N°	N° (%)	Mean ± SD (Range), y.o	N°	N°	Mean ± SD (Range), months
Goksan (1992) [22]	IV	6	19 / 18	1 (5.2%)	61.4 (42–74)	6	12	60
Whiteside (2011) [23]	IV	12	18 / 18	0 (0%)	69 ± 6	7	11	62 (27–96)
Singer (2012) [24]	IV	10	72 / 63	9 (12.5%)	70.7 ± 10.5	31	32	36 (24–70)
Baker (2013) [25]	III	15	33 / 33	0 (0%)	69.4 ± 10.7	18	15	14
Jenny (2013) [26]	IV	12	47 / 47	0 (0%)	72 (45–93)	20	27	36
Tibrewal (2014) [27]	IV	10	50 / 50	0 (0%)	66.8 (42–84)	17	33	126 (12–240)
Haddad (2015) [28]	III	14	28 / 28	0 (0%)	63 (48–87)	14	14	78 (36–108)
Massin (2016) [29]	III	16	108 / 108	0 (0%)	71 (63–76)	53	55	44 ± 25
Zahar (2016) [30]	IV	8	70 / 59	11 (15.7%)	70 (60–81)	39*	31*	120 (102–132)
Castellani (2017) [31]	III	13	35 / 35	0 (0%)	68 (61–79)	16	19	16 (12–25)
Abdelaziz (2019) [32]	IV	17	91 / 72	19 (20.8%)	70 ± 8.2	41	31	49.9 ± 18.8
Holland (2019) [33]	IV	12	31 / 25	6 (19.4%)	72.3 ± 7.4	N/A	N/A	36 (24–102)
Klemt (2020) [34]	IV	16	53 / 44	9 (16.9%)	65.1 ± 9.4	28*	25*	21.4 ± 4.9
Ji (2021) [35]	IV	11	160 / 132	28 (17.5%)	68.6 (40–86)	34	98	51.6 (24–85)
Pellegrini (2021) [36]	IV	9	20 / 20	0 (0%)	67.6 ± 10.2	9	11	74.4 (24–120)
Razii (2021) [37]	IV	10	84 / 80	4 (4.7%)	68 (36–92)	53*	31*	84 (12–144)
Rossman (2021) [38]	IV	5	57 / 40	17 (29.8%)	68 ± 9	24	16	80 (22–172)
Tuecking (2021) [39]	IV	7	15 / 15	0 (0%)	65 ± 10.2	4	11	47.3 ± 19.2
Overall	IV	/	991/887	104 (10.5%)	67.9 ± 2.9	294 (43.3%)	385 (56.7%)	57.6 ± 31.8

*N*° Number of evaluation cases, *LoE* Levels of evidence, *MINORS* score Methodological index for non-randomized studies score, % Percentage, *SD* Standard deviation, *y.o* Years old, *N/A* Not available. *Sex data refer to the final patient cohort, except for these three studies that reported only data from the initial patient cohort

### Reinfection after one-stage revision

All eighteen studies reported overall failure due to infection after one-stage revision [22–39]. Reinfection after one-stage revision occurred in 108 cases (12.2%). Survivorship after the one-stage revision was 87.8% (779 patients were infection-free at the last follow-up). The mean time between one-stage revision and reinfection was 26.7 ± 14.4 months as reported by ten studies [22–24, 27, 32, 33, 35, 37–39]. Thirteen studies [22–24, 26–30, 32, 35, 37–39] reported the treatment performed for reinfection. A repeated one-stage revision was the most frequent intervention, followed by DAIR, long-term antibiotic therapy, knee arthrodesis, AKA, and two-stage revision. The survivorship after treatment for reinfection considering failure also patients who underwent AKA or knee arthrodesis was 92.1% (817 patients). Data on reinfection after one-stage revision, success, and failure rate of treatments for reinfections, and survivorship are listed in Table 2.

**Table 2:** Reinfection after one-stage revision, treatment for reinfection and survivorship after reoperation

Author and publication year	N° of knees	Reinfection after one-stage revision		Treatment for reinfection after one-stage revision						Infection-free survival at final follow-up	
		N°	%	One-stage revision N° (success/failure)	DAIR N° (success/failure)	Long-term antibiotic therapy N°(success/failure)	Two-stage revision N° (success/failure)	Knee arthrodesis N°	AKA N°	N°	%
Goksan (1992) [22]	18	2	11,1%	1 (1/0)	0	1 (0/1)	0	0	0	17	94,4%
Whiteside (2011) [23]	18	1	5,6%	0	1 (1/0)	0	0	0	0	18	100,0%
Singer (2012) [24]	63	3	4,8%	0	0	0	0	3	0	60	95,2%
Baker (2013) [25]	33	7	21,2%	N/A	N/A	N/A	N/A	N/A	N/A	26	78,8%
Jenny (2013) [26]	47	6	12,8%	2 (2/0)	0	3 (1/2)	1 (0/1)	0	0	44	93,6%
Tibrewal (2014) [27]	50	4	8,0%	0	2 (2/0)	1 (1/0)	0	1	0	49	98,0%
Haddad (2015) [28]	28	0	0,0%	0	0	0	0	0	0	28	100,0%
Massin (2016) [29]	108	25	23,1%	11 (6/5)	7 (7/0)	3 (0/3)	0	2	2	96	88,9%
Zahar (2016) [30]	59	3	5.1%	3 (2/1)	0	0	0	0	0	56	94,9%
Castellani (2017) [31]	35	2	5,7%	N/A	N/A	N/A	N/A	N/A	N/A	33	94,3%
Abdelaziz (2019) [32]	72	8	11,1%	6 (6/0)	0	0	0	0	2	70	97,2%
Holland (2019) [33]	25	1	4,0%	N/A	N/A	N/A	N/A	N/A	N/A	24	96,0%
Klemt (2020) [34]	44	11	25,0%	N/A	N/A	N/A	N/A	N/A	N/A	33	75,0%
Ji (2021) [35]	132	9	6,8%	1 (1/0)	4 (4/0)	2 (2/0)	0	0	2	130	98,5%
Pellegrini (2021) [36]	20	0	0,0%	0	0	0	0	0	0	20	100,0%
Razii (2021) [37]	80	7	8.75%	2 (2/0)	2 (1/1)	1 (1/0)	1 (1/0)	1	0	77	96,3%
Rossman (2021) [38]	40	15	37,5%	N/A	N/A	N/A	N/A	1	2	25	62,5%
Tuecking (2021) [39]	15	4	26,7%	N/A	N/A	N/A	N/A	N/A	N/A	11	73,3%
Overall	887	108	12.2%	26 (20/6)	16 (15/1)	11 (5/6)	2 (1/1)	8	8	817	92,1%

*N*° Number of evaluation cases, % Percentage, *DAIR* Debridement, antibiotics, and implant retention, *AKA* Above-knee amputation, *N/A* Not available

### Clinical outcomes after one-stage revision

Seven studies reported the mean postoperative range of motion (ROM) [22, 24, 26, 29, 32, 33, 36]. Six studies reported the mean preoperative and postoperative knee society score (KSS) knee score [23, 24, 26, 28, 29, 36]. One study reported a mean preoperative KSS function score [24], whereas the mean postoperative KSS function score was reported by three studies [24, 26, 29]. Two studies reported the mean preoperative Oxford knee score (OKS) [27, 37], and six studies reported the mean postoperative OKS [24, 25, 27, 29, 33, 37]. Three studies reported the mean preoperative Hospital for Special Surgery (HSS) score [30, 32, 35], and three studies reported the mean postoperative HSS [30, 32, 35]. For all the clinical scores, there was a statistically significant improvement (*p* < 0.05) between the preoperative and postoperative mean scores (Table 3).

**Table 3:** Clinical outcomes after one-stage revision

Clinical outcomes after one-stage revision	Preoperative values	Postoperative values	*P* value
	Mean value ± SD/Range	Mean value ± SD/Range	
ROM	N/A	96.6° ± 6.3 (90.4°-104°)	**//**
KSS knee score	27.3 ± 7.8	81.5 ± 6.3	** < *0.05***
KSS function score	21	74.2 ± 3.5	** < *0.05***
OKS	34.2 ± 2.9	61.6 ± 6.1	** < *0.05***
HSS score	17.9 ± 6.7	32.2 ± 4.6	** < *0.05***

*SD* Standard deviation, *ROM* Range of motion, *N/A* Not available, degrees, *KSS* Knee society score, *OKS* Oxford knee score, *HSS* Hospital for special surgery

### Microbiology

Sixteen studies reported data on the causative microorganism at the one-stage revision [22–24, 26–33, 35, 39]. Gram-positive bacteria caused infection in 71.1% of cases, gram-negative bacteria in 7.5% of cases, and a polymicrobial infection in 8% of cases. No bacterial growth was reported in 9% of cases. Fungal infection occurred in 0.8% of cases. Table 4 shows detailed information on the causative microorganisms at the time of the one-stage revision.

**Table 4:** Causative microorganism at the one-stage revision

		Gram-positive									Gram-negative						Others			
Author and publication year	N° of causative microrganism	MRSA/ MRSE	Enterococcus spp	S. Aureus	S. Epidermidis	CoNS	Other Staphylococcus spp.	Streptococcus spp.	P. Acnes	Others	Klebsiella Pneumonia	Enterobacter	Pseudomonas	E. Coli	Proteus	Others	Fungal	Polym	NG	NS
	N° / N°	N° (%)	N° (%)	N° (%)	N° (%)	N° (%)	N° (%)	N° (%)	N° (%)	N° (%)	N° (%)	N° (%)	N° (%)	N° (%)	N° (%)	N° (%)	N° (%)	N° (%)	N° (%)	N° (%)
Goksan (1992) [22]	18	0	0	6	8	0	0	0	0	0	0	0	0	0	0	0	0	2	2	0
Whiteside (2011) [23]	18	18	0	0	0	0	0	0	0	0	0	0	0	0	0	0	0	0	0	0
Singer (2012) [24]	63	0	6	15	19	0	15	3	5	0	0	0	0	0	0	0	0	0	0	0
Baker (2013) [25]	N/A	N/A	N/A	N/A	N/A	N/A	N/A	N/A	N/A	N/A	N/A	N/A	N/A	N/A	N/A	N/A	N/A	N/A	N/A	N/A
Jenny (2013) [26]	49	5	1	10	9	0	0	5	0	0	0	0	2	0	0	0	0	0	15	2
Tibrewal (2014) [27]	50	2	0	16	15	0	0	0	0	0	0	0	0	0	0	0	0	9	0	8
Haddad (2015) [28]	28	0	0	8	0	11	0	4	0	0	0	0	0	0	0	5	0	0	0	0
Massin (2016) [29]	108	9	0	33	0	29	0	14	0	12	0	0	0	0	0	10	0	0	1	0
Zahar (2016) [30]	59	9	2	4	14	9	0	7	0	2	0	5	0	0	0	3	1	3	0	0
Castellani (2017) [31]	35	0	1	4	0	11	0	2	0	0	0	0	0	0	0	1	0	3	0	13
Abdelaziz (2019) [32]	72	1	3	9	28	0	8	11	0	0	1	0	1	2	1	0	0	0	4	3
Holland (2019) [33]	N/A	N/A	N/A	N/A	N/A	N/A	N/A	N/A	N/A	N/A	N/A	N/A	N/A	N/A	N/A	N/A	N/A	N/A	N/A	N/A
Klemt (2020) [34]	44	1	0	7	0	3	6	7	0	2	0	0	1	0	0	3	0	6	6	2
Ji (2021) [35]	132	25	2	21	19	0	2	0	0	5	9	4	3	6	2	1	5	1	27	0
Pellegrini (2021) [36]	20	2	1	6	0	9	0	0	1	0	0	0	0	0	0	0	0	0	0	1
Razii (2021) [37]	80	3	1	8	0	28	0	3	0	1	0	0	1	0	1	1	0	21	16	0
Rossman (2021) [38]	40	0	19	0	0	0	0	0	0	0	0	0	0	0	0	0	0	21	0	0
Tuecking (2021) [39]	15	0	1	0	0	5	2	0	1	0	0	0	0	0	0	0	1	1	4	0
Overall	835	75 (9%)	37 (4.4%)	147 (17.6%)	112 (13.4%)	105 (12.6%)	33 (4%)	56 (6.7%)	7 (0.8%)	22 (2.6%)	10 (1.2%)	9 (1.1%)	8 (1%)	8 (1%)	4 (0.5%)	24 (2.9%)	7 (0.8%)	67 (8%)	75 (9%)	29 (3.4%)

*MRSA* Methicillin-resistant Staphylococcus aureus, *MRSE* Methicillin-resistant staphylococcus epidermidis, *spp* Species, *S. Aureus* Staphylococcus Aureus, *S. Epidermidis* Staphylococcus Epidermidis, *CoNS* Coagulase-negative staphylococci, *P. Acnes* Propionibacterium Acnes, *E. Coli* Escherichia Coli, *Polym* Polymicrobial infection, *NG* No culture growth, *N/A* Not available, *N*° Number of evaluation cases, %: percentage

Thirteen studies reported data on the causative microorganism at reinfection [22, 23, 26–28, 30, 32, 33, 35– 39]. The causative microorganisms were gram-positive in 44.4% of cases, gram-negative in 11.2%, and other microorganisms in 44.4%. Table 5 shows detailed information on the causative microorganisms at the time of the reinfection.

**Table 5:** Causative microorganism at the reinfection

Author and publication year	N° of causative microrganism	Gram positive	Gram-negative	Others microorganisms
	N°	N° (%)	N° (%)	N° (%)
Goksan (1992) [22]	2	1	1	0
Whiteside (2011) [23]	1	1	0	0
Singer (2012) [24]	N/A	N/A	N/A	N/A
Baker (2013) [25]	N/A	N/A	N/A	N/A
Jenny (2013) [26]	6	2	4	0
Tibrewal (2014) [27]	4	0	0	4
Haddad (2015) [28]	0	0	0	0
Massin (2016) [29]	5	1	0	4
Zahar (2016) [30]	N/A	N/A	N/A	N/A
Castellani (2017) [31]	8	8	0	0
Abdelaziz (2019) [32]	1	0	0	1
Holland (2019) [33]	N/A	N/A	N/A	N/A
Klemt (2020) [34]	9	3	1	5
Ji (2021) [35]	0	0	0	0
Pellegrini (2021) [36]	8	2	0	6
Razii (2021) [37]	15	10	1	4
Rossman (2021) [38]	4	0	0	4
Tuecking (2021) [39]	N/A	N/A	N/A	N/A
Overall	63	28 (44.4%)	7 (11.2%)	28 (44.4%)

*N* Number of evaluation cases, % Percentage, *N/A* Not available

In the one-stage revisions, 67.8% “favorable” microorganisms and 32.2% “aggressive” microorganisms were identified. Methicillin-resistant Staphylococcus Aureus (MRSA), methicillin-resistant Staphylococcus Epidermidis (MRSE), Enterococcus species, Pseudomonas aeruginosa, Fungal, polymicrobial infection, and culture-negative PJI were considered “aggressive” microorganisms. Reinfection was caused by “favorable” microorganisms in 44.4% of cases and by an “aggressive” microorganism in 55.6% of cases. Gram-positive infections were significantly higher at one-stage revision than at reinfection (71.1% and 44.4%, respectively, *p* < 0.001). Among gram-positive infections, there was a significant difference for Staphylococcus Epidermidis (13.4% at one-stage revision and 4.8% at reinfection, *p* = 0.047) and coagulase-negative staphylococci (CoNS) (12.6% at one-stage revision and 3.2% at reinfection, *p* = 0.026). No statistical differences were found between one-stage revision and reinfection for the overall gram-negative infections (7.5% and 11.1%, respectively, *p* = 0.308). Analyzing the individual gram-negatives showed that infections caused by Pseudomonas Aeruginosa (1% at one-stage revision and 4.8% at reinfection, *p* = 0.008) and Escherichia Coli (1% at one-stage revision and 4.8% at reinfection, *p* = 0.008) were more frequent at reinfection with a statistically significant difference (Table 6).

**Table 6:** Differences in microbiology between one-stage revision and reinfection

Gram-positive bacteria					
Microorganisms	One-stage revision		Reinfection		*p*
	N°	%	N°	%	
MRSA *	56	6.7%	4	6.3%	0.912
S. Aureus	147	17.6%	9	14.3%	0.502
MRSE *	19	2.3%	1	1.6%	0.721
S. Epidermidis	112	13.4%	3	4.8%	***0.047***
CoNS	105	12.6%	2	3.2%	***0.026***
Staphylococcus spp.	33	4%	2	3.2%	0.758
Streptococcus spp.	56	6.7%	3	4.8%	0.548
P. Acnes	7	0.8%	0	0%	N/A
Enterococcus spp. *	37	4.5%	4	6.3%	0.481
Others gram-positive	22	2.6%	0	%	N/A
Overall	594	71,1%	28	44,4%	** < *0.001***
Gram-negative bacteria					
Klebsiella Pneumonia	10	1.2%	1	1.6%	0.786
Enterobacter spp.	9	1.1%	N/A	N/A	N/A
Pseudomonas Aeruginosa *	8	1%	3	4.8%	***0.008***
E. Coli	8	1%	3	4.8%	***0.008***
Proteus Mirabilis	4	0.5%	0	0%	N/A
Others gram-negative bacteria	24	2.8%	0	0%	N/A
Overall	63	7,5%	7	11,1%	0.308
Other microorganisms					
Fungal *	7	0.8%	5	7.9%	** < *0.001***
Polymicrobial Infection *	67	8,0%	11	17.5%	***0.010***
NG *	75	9,0%	7	11.1%	** < *0.001***
NS	29	3.5%	5	7.9%	N/A
Microorganisms’ aggressiveness					
Nonaggressive	566	67,8%	28	44,4%	** < *0.001***
Aggressive *	269	32,2%	35	55.6%	** < *0.001***

*N* Number of evaluation cases, % Percentage, *N/A* Not available, *MRSA* Methicillin-resistant Staphylococcus aureus, *MRSE* Methicillin-resistant staphylococcus epidermidis, *spp* Species, *S. Aureus* Staphylococcus Aureus, *S. Epidermidis* Staphylococcus Epidermidis, *CoNS* Coagulase-negative staphylococci, *P. Acnes* Propionibacterium Acnes, *E. Coli* Escherichia Coli, *NG* No culture growth, *NS* Not specified, *: these are microorganisms that are considered aggressive

## Discussion

PJI represents one of the most challenging complications after TKR [1, 2]. For many years, the two-stage revision was considered the gold-standard procedure; however, recently, single-stage treatment has gained popularity [8, 9]. The main result of this study is that one-stage revision, in line with data reported by previous studies, provides an equivalent or slightly lower reinfection rate than two-stage revision [40, 41]. Furthermore, the success rate from reinfection reoperation was lower than the first revision surgery for an infection. Finally, it was demonstrated that causative microorganisms differ significantly between the first one-stage revision and infection recurrence.

### Infection recurrence

The reinfection recurrence rate reported after the one-stage revision was 12.2% at a mean time of 26.7 months, ranging from 0 [28, 36] to 37.5% [38]. This result is comparable to the reinfection rate described in the literature with two-stage revision, which varied between 10 and 30% [40]. Rossman et al. [38] reported the highest reinfection rate among the studies included in this systematic review. The authors observed reinfection in 15 of 40 enterococcal-related PJIs. Castellani et al. [31] reported similar data with a reinfection rate of 50% in enterococcal-related PJIs in patients treated with one- or two-stage revision. Citak et al. [42] highlighted enterococci, being difficult-to-treat antibiotic-resistant pathogens, as an independent risk factor for reinfection in PJI, regardless of the surgical treatment performed. Antibiotic-resistant bacteria were one of the main topics of the second international meeting on PJI, whose guidelines did not recommend one-stage revision in case of systemic signs of infection, an infection caused by a resistant microorganism, culture-negative infection, and insufficient soft tissue coverage [43]. The higher revision rates described in some of the studies included in this systematic review [32, 38, 42] may be explained by a one-stage revision approach even in the presence of the above-mentioned risk factors [43].

### Reoperations

In this systematic review, treatments performed for infection recurrence (71 PJIs) were examined. No infection at final follow-up was found in 92.1% of patients. A further one-stage revision was the most frequent treatment for reinfection. It was performed on 26 patients with a success rate of 76.9%. DAIR was reported in 16 cases, with a success rate of 93.8%. Lower results were observed in the 11 patients treated exclusively with suppressive antibiotic therapy and the two patients who underwent two-stage revision, with success rates of 45.5% and 50%, respectively. “Salvage procedures” were performed in 16 cases; eight patients underwent knee arthrodesis, while AKA was necessary in eight cases.

### Microbiology

This study highlighted that the causative microorganisms at one-stage revision and reinfection were significantly different (Table 5). The incidence of gram-positive bacteria, especially Staphylococcus epidermidis and CoNS, decreased significantly from 71.1% at one-stage revision to 44.4% at reinfection. Gram-negative bacteria were more frequently in reinfection (11.1%) than in one-stage revision (7.5%), but not at a statistically significant rate, except for Pseudomonas Aeruginosa and Escherichia Coli, which both increased from 1 to 4.8%, respectively. Finally, polymicrobial, culture-negative, or fungal infections, often considered challenging PJI, had a statistically higher reinfection rate (Table 6). The results presented in this systematic review follow the data reported in the literature on the increased reinfection rate after surgical treatment of knee PJI caused by “aggressive” microorganisms [43–45]. Specifically, this study observed that infections caused by “aggressive” species had a statistically significant higher incidence of reinfection after one-stage revision [43–45].

PJI caused by resistant staphylococci, MRSA and MRSE, is considered a major therapeutic challenge with a high rate of recurrent infection. Mittal et al., in their series of 37 patients, reported reinfection in 24% of patients [46]. Salgado et al., in their retrospective study, observed a 50% reinfection rate in MRSA-related infections [47]. In this systematic review, PJI was caused by resistant staphylococci in 12.7% of patients. MRSA caused recurrent infection in four cases (6.3%) and MRSE in one case (1.6%). Generally, PJI caused by resistant staphylococci is treated with a two-stage revision, considering the higher chances of eradication of the infection due to the debridement of the first stage and the high concentration of local antibiotic released by the antibiotic from the cement spacer [48]. Although some authors have considered resistant staphylococcal infections a contraindication to single-stage revision [28, 36], the results of this paper suggest that one-stage revision could be performed with favorable results even in PJIs caused by resistant staphylococci. In this systematic review, only one study reported a complete series of patients affected by MRSA PJI [23]. All patients underwent a one-stage revision followed by intra-articular injection of antibiotic (500 mg Vancomycin) once or twice daily for six weeks without postoperative intravenous antibiotics. The authors reported a 94.4 percent success rate (17 of 18 patients). In the only case of failure, the patient underwent reoperation after five months with a large fragment of necrotic bone removal [23].

Several authors have considered fungal infection a contraindication for one-stage revision suggesting two-stage revision as a gold-standard procedure [49–51]. In a recent systematic review of the literature [49] that collected data from 45 fungal knees PJIs, it was demonstrated that the causative microorganism responsible for about 80 percent of the infections was the Candida species. About 50 percent of the patients had risk factors such as advanced immunodepression, prolonged antibiotic use, autoimmune disease, or drug abuse. After a mean follow-up of 37.4 months, the authors reported recurrent bacterial infections in five cases. Instead, six patients required AKA. In another six cases, no established information on therapeutic outcomes was reported [49]. In this systematic review, seven patients with fungal PJI underwent one-stage revision with a failure rate of 42.8%. Fungal PJI should always be suspected in case of infection recurrence. This paper reported fungal PJI in five cases after a one-stage revision procedure.

Polymicrobial infections have been considered an independent risk factor for infection recurrence following several surgical treatments for PJI [16, 52, 53]. Razii et al. reported that polymicrobial infections were associated with increased reinfection [37]. Similarly, Massin et al. observed a higher recurrent infection rate in patients with polymicrobial infections caused by gram-negative bacteria [29]. Two of the studies included in this systematic review reported a higher risk of reinfection in polymicrobial infections [27, 37]. Several authors agreed that polymicrobial infection is a contraindication for one-stage revision, suggesting that other surgical treatments, such as two-stage revisions, are more suitable for this clinical condition [28, 33, 39].

### Limitations

This systematic review has some limitations that need to be analyzed. First, only English studies were considered, which may exclude any relevant studies published in other languages. Second, the LoE of the included papers is poor, and there are no studies with LoE I or II. Third, there is significant variability among studies regarding PROMs and outcome measures evaluated. Finally, the different papers have various inclusion criteria for patients eligible for one-stage revision, and the patients’ populations are difficult to compare.

## Conclusions

This systematic review reported that, in selected patients, the incidence of recurrent infection after one-stage revision is comparable to or lower than that of other surgical treatments such as two-stage revision and DAIR. Reoperation for recurrent infection demonstrated a lower success rate than the first one-stage procedure. In addition, microbiology is significantly different between one-stage revision and reinfection. Higher rates of “aggressive” microorganisms have been observed in the latter. Finally, several risk factors should be evaluated before performing a one-stage revision. In this systematic review, it was reported that in the presence of previous septic events and aggressive and polymicrobial infections, the risk of reinfection is higher, and other surgical strategies, such as two-stage revisions, should be considered.

## Data Availability

Dataset analyzed in this study is available from the corresponding author on reasonable request.
